# Autonomous pump against concentration gradient

**DOI:** 10.1038/srep23414

**Published:** 2016-03-21

**Authors:** Zhi-cheng Xu, Dong-qin Zheng, Bao-quan Ai, Wei-rong Zhong

**Affiliations:** 1 Siyuan laboratory, Guangzhou Key Laboratory of Vacuum Coating Technologies and New Energy Materials, Department of Physics, Jinan University, Guangzhou 510632, China; 2 Laboratory of Quantum Engineering and Quantum Materials, ICMP and SPTE, South China Normal University, Guangzhou 510006, China

## Abstract

Using non-equilibrium molecular dynamics and Monte Carlo methods, we have studied the molecular transport in asymmetric nanochannels. The efficiency of the molecular pump depends on the angle and apertures of the asymmetric channel, the environmental temperature and average concentration of the particles. The pumping effect can be explained as the competition between the molecular force field and the thermal disturbance. Our results provide a green approach for pumping fluid particles against the concentration gradient through asymmetric nanoscale thin films without any external forces. It indicates that pumping vacuum can be a spontaneous process.

In 1994, Satller’s group^1^ had found the nanocone formed by carbon through STM. Krishnan A. etc.^2^ reported generally on the graphitic cones and the nucleation of curved carbon surfaces in 1997. Since then many new phenomena have been discovered and the theoretic research on the mechanical, electrical and thermal properties of carbon nanocone have been extensively studied^3^,^4^. In 2008, G. K. Dimitrakakisand co-workers^5^ proposed a three-dimensional network of nano-structural model in which the nanotube formed by the combination of five-carbon rings and seven-carbon rings transformed to graphite to enhance its capacity of hydrogen storage. In 2010, González team^6^ gave a comprehensive theoretic analysis of the analogous structures. In the last two decays, mass transport and diffusion in nanostructure caused more and more concerns^7^,^8^,^9^,^10^,^11^,^12^,^13^. Besides, the bioactivities in a living system, such as the cellular transport, synthesis of ATP, cell division, signal transduction and muscle contraction, are inevitably involved with the molecular transport in various nanotubes where the aperture of the tube will more or less vary in size. In the structure of Aquaporin-1, one can testify that the aperture of the nanotube gradually changes and the tube is horn-shaped with a minimum aperture of 0.38 *nm*^14^. Therefore, the nanochannels with varying aperture are ubiquitous in nanoscale.

As for the molecular transport inasymmetric nanotubes, a number of researchers have conducted numerous investigations both in theory and experiment and found many interesting phenomena. In 1986, Tsong *et al.*^15^,^16^ usedratchet effect to explain the directed transport in organisms and further connected it with molecular pump. In 2008,Chinappi M. etc.^17^,^18^ testified the ratchet effect in molecular transport in asymmetric nanochannels through molecular dynamics simulation. In 2009, Li^19^ had observed that asymmetric nanochannels have the function of sieving molecules. According to the previous results, one can utilize various kinds of driving force such as chemical gradient^20^, thermal gradient^21^,^22^, osmotic pressure^23^, point charge and alternating electric field^24^,^25^,^26^, to realize the molecular pump. Meanwhile, in terms of extremely small scales, the asymmetric nanochannels, which shows distinguished asymmetric distribution of force field and molecular transport on the surface of the channels, has a clear impact on the fluid molecules. Liu *et al.*^27^ have used electric field to transport ion through an asymmetric biological channel. Therefore, the asymmetric nanochannels in extremely small size could be constructed into new molecular pump, providing a new approach for transforming other kinds of energy into mechanical energy for human activities.However, whether an asymmetric channel can pump particle against the concentration is still not clear.

According to thermodynamic theories, if the chemical potential is uniform in the system, no molecule transport should be expected. Actually, the asymmetric liquid and ion current rectification are also observed in recent experiments^28^,^29^,^30^,^31^. Thepressure differences for the convergent and divergent directions are studied in conical channel through molecular dynamic simulations^32^, it is reported that the convergent water flux is changed fromsmallerto larger than the divergent one with the increase of pressure.It seems that the thermodynamic theory becomes inapplicable in nanoscale system. It is usually explained as a net flux induced by thermal noise, where its correlation timelength is large for the nanoscale system^33^.

In this work, we will show an asymmetric nanochannel that can pump particles from low density areas to high density areas. We will also investigate the following aspects that can affect the pumping efficiency: the angle of the nanocone, the atomic attraction, the aperture of the cone, the temperature, the density and the type of atoms. Moreover, we will suggest the physical mechanism of the molecular pump. This may provide a new way for studying the molecular transport in bionics and organism. The asymmetric thin films may be a green structure for molecular transport.

## Model and Methods

**Figure 1:**
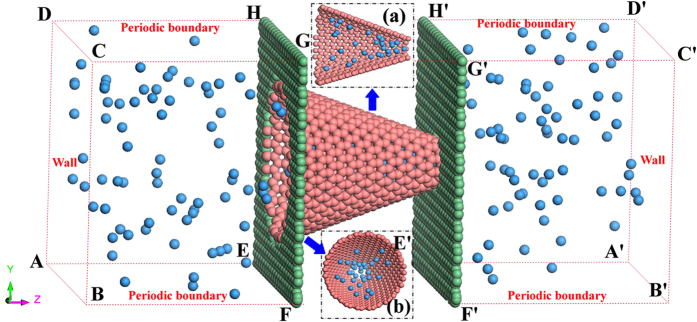
Diagram of the asymmetric carbon nanochannels; the insets (**a,b**) are side section and cross section of the nanotube, respectively.

Figure 1 shows the diagram of the asymmetric nanotubes. The left box represents the left atom reservoir with the size of *AE* × *AB* × *AD* = 4.6 *nm* × 4.6 *nm* × 4.0 *nm*, the blue balls stand for the fluid particles and the green ones for the carbon atoms. The plane *ABCD* is set as the rigid wall, plane *EFHG* as the partition of graphene, and the rest four planes are the periodic boundaries. The right box represents the right atom reservoir with the size of *A’E’* × *A’B’* × *A’D’* = 4.6 *nm* × 4.6 *nm* × 4.0 *nm*, the blue balls stand for the rare gases and the green ones for the carbon atoms. The plane *A’B’C’D’* is set as the rigid wall, plane *E’F’H’G’*as the partition of graphene, and the rest four planes are the periodic boundaries. The carbon nanocones with the length *L*ASC = 3.0 *nm* (also the distance between the plane *EFHG* and *E’F’H’G’*) connect the left reservoir with the rightreservoir. The properties of the object transporting in the nanochannels can be studied by changing the aperture and angle of the asymmetric nanochannels which is represented by the pink balls. We use Monte Carlo method to control the number of atoms in the two atom reservoirs in which the fixed absolute activity is used to adjust^34^,^35^. When the absolute activity is less (or bigger) than the preset value *z*, the system will automatically create (or delete) an atom. Creations are accepted with probability


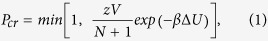


where 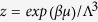 is the absolute activity at temperature 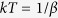, *μ* is the chemical potential, Λ is the de Broglie wavelength, i.e. 
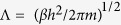
, *V* is the volume of the left or right reservoir, *N* represents the number of atoms in left or right reservoir, and Δ*U* is the variation of potential energy after the addition or destruction of an atom. When a creation is accepted, the atom is given a velocity, selected from Maxwell-Boltzmann distribution according to the simulation temperature. Meanwhile, destructions are accepted with probability


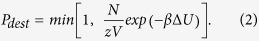


In this paper, we choose the asymmetric carbon nanotubes as the transport medium for its mature industrial production and extensive investigation, and the rare gases helium (He) and neon (Ne) as the transport object for their chemical steadies. In the simulations, non-equilibrium molecular dynamics method is utilized, and the four different interactions between He and He, Ne and Ne, He and C, Ne and C are described by the Lennard-Jones potential^36^. In the whole process, the position of carbon atoms is fixed. The temperature is set according to the Langevin random thermostatwith a step of 0.55 fs. In order to collect the particle current rapidly and precisely, we set different equilibrium time in the system depending on different circumstances and calculate the particle current after the equilibrium.

Usually, if the chemical potential of left atom reservoir is larger than that of the right atom reservoir, the atoms will flow out the left reservoir, go through the middle channel and flow into the right reservoir in the end. Therefore, the system as shown in Fig. 1 is a nonequilibrium grand canonical ensemble. Here the mass flux also reflects the pumping ability of asymmetric carbon nanotubes. We define the pump efficiency as the mass flux J, which means the molar mass per unit cross area and time. The mass flux *J* is


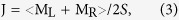


where M_L_, M_R_ are the total molar mass flow out the left reservoir or flow into the right reservoir, respectively. *S* is the average cross area of the channel. <> means the time average. In our simulations, the simulated time is about 10^9^ steps or 550 *ns*, which is long enough for the system to evolve into a steady state.

## Analyzing the asymmetric channel

**Figure 2:**
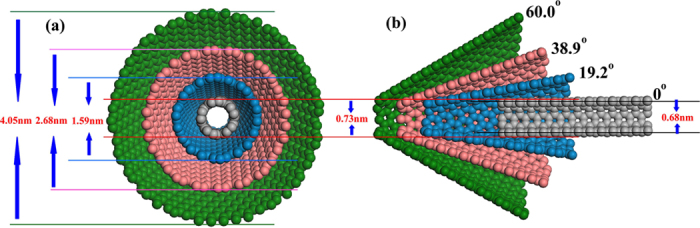
Diagrams of asymmetric carbon nanotubes with various angles, (**a**) the cross section, (**b**) side section.

**Figure 3:**
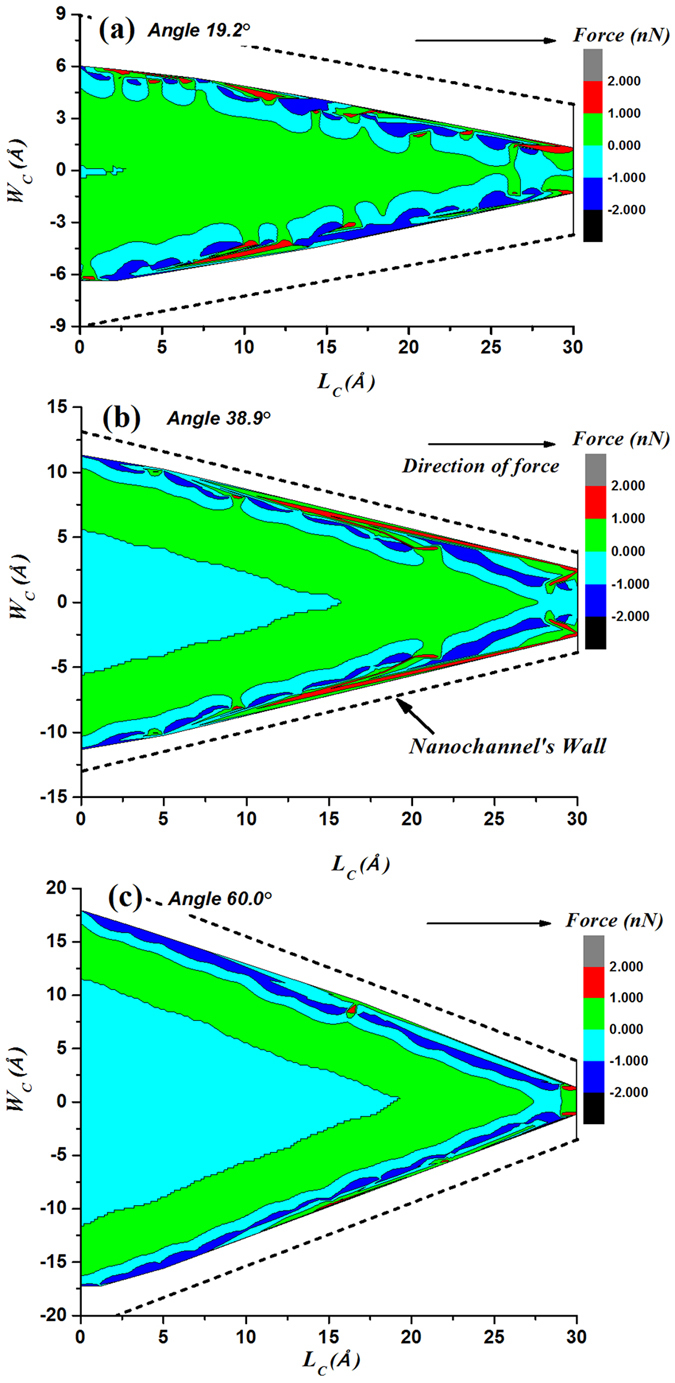
Force distribution in the asymmetric nanotubes with different angles: (**a**) 19.2°, (**b**) 38.9°, and (**c**) 60.0°. The dash lines are the walls of nanochannels.

Considering that the equilibrium position of Lennard-Jones potential between He and C is 0.298 *nm*, we need to construct proper resultant force at the small aperture of the asymmetric nanotube. Therefore, we choose the asymmetric nanotube with a small aperture of 0.73 *nm* as our object for the analysis of structure. As it is shown in Fig. 2, with the same small aperture of 0.73 *nm*, the bigger the angle means the wider the big aperture of the asymmetric nanotube, which is defined as the intersection angle between the hypotenuse of the asymmetric nanochannel and the symmetry axis,. In order to clearly describe the force distribution along *Z* axis in the asymmetric nanotubes, we use helium as the probing atom and calculate the force distribution with angle 19.2°, 38.9° and 60.0° in the asymmetric channel, which is shown in detail in Fig. 3, the horizontal axis is the direction of the varying length of the asymmetric channel, the vertical axis is the direction of the varying breadth of the channel and the arrow is the positive direction of the force field. Comparing the subfigures in Fig. 3, one can find that with the increase of the angle, the area of the positive force helium can experience in the asymmetric channel is decreasing, and the area of zero-force is expanding rapidly. Aside from this, there exists a 0.3 *nm* long area of negative force at the small aperture in different asymmetric channels, which means the potential barrier appears. However, in general, helium is obviously affected by the area of positive force in the system of extremely small size, which will drive helium flowing into the smaller aperture.

## Testing conception

**Figure 4:**
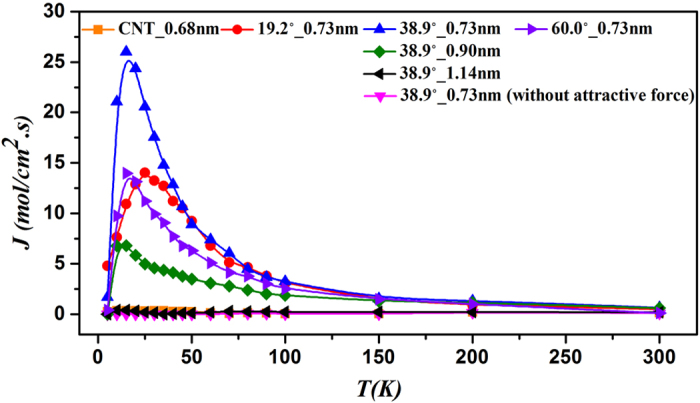
Temperature dependence of the unit diffusion flux of He in different asymmetric nanotubes.

To testify whether the above-mentioned model has the molecular pump effect or not, we investigate the model in Fig. 1 and select three types of carbon nanotubes as the transport channels whose angles are 19.2°, 38.9°and 60.0°, respectively, and the symmetric carbon nanotube (5,5) with a diameter of 0.68 *nm* as the comparison group. In this process of simulation, the length of the transport channel is 3.0 *nm*, and the density of both the left and right atom reservoirs is 0.0015 *mol/cm*^3^. Usually, due to the same concentration between the left and right particle reservoirs, the particle current should be zero. As shown in Fig. 4, symmetric carbon nanotubes of extremely small size show no pump effect at any temperature. However, surprisingly the asymmetric nanotubes with angle of 19.2°, 38.9° and 60.0° show pump effect, especially for that of 38.9°. If the aperture is larger than 1.14 nm, the directional transport phenomenon will disappear.

With the increase of temperature, the pumping efficiency of the three channels shows a maximum value at certain temperature. The value of the peak will move to lower temperature range with the increase of the angle. It is well known that the kinetic energy of particle comes mainly from thermal fluctuations. In the low temperature region, the particles have not enough kinetic energy, and then the current is small. When the temperature increases, the kinetic energy increases. Therefore, the particle current increases. However, if the temperature increases to large enough, the asymmetry of the nanochannel will be weaken by thermal fluctuation. Accordingly, the particle current decreases.

To testify whether this directional transport phenomenon is caused by the force field, we eliminate the attraction part of (*σ*/*r*)^6^ in the Lennard-Jones potential and retain the repellant part of (*σ*/*r*)^12^. As one can see in Fig. 4, no directional transport phenomenon appears. Therefore, the force field of asymmetric channel in small size motivates the directional transport where the attraction force plays an important role.

**Figure 5:**
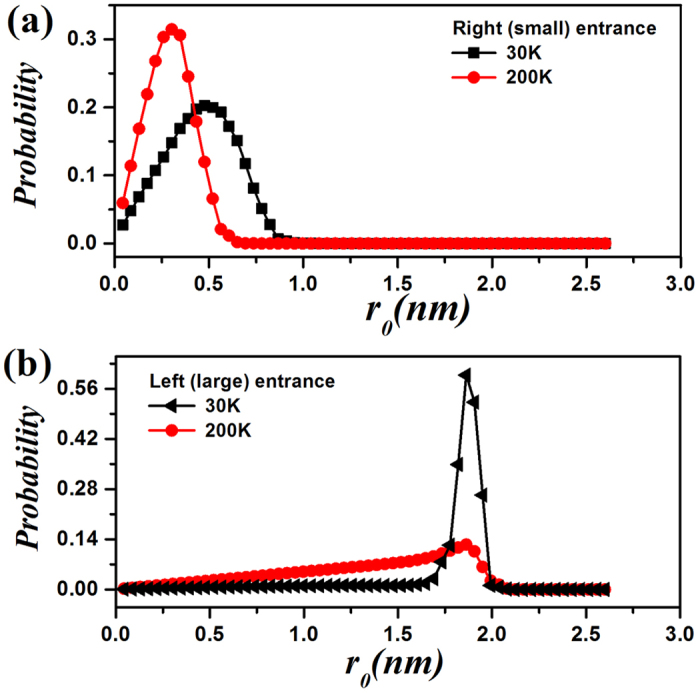
The probability distribution of atoms at the right (**a**) and left (**b**) entrance.

To find out exactly how the asymmetric force field affect the movement of helium in the channel, we record the position distribution of helium with various apertures at the entrance of the channel with the cone angle of 38.9° which is shown in Fig. 5. It can be noticed that He tends to be attracted to the wall of asymmetric channel with the decrease of temperature no matter where the entrance is, and the detailed information can be retrieved in the insets (a) and (b) in Fig. 1. It can be concluded that in the area of positive field more atoms will overcome the thermal disturbance at low temperature or at low speed and then show a directional transport phenomenon, i.e. helium pump.

**Figure 6:**
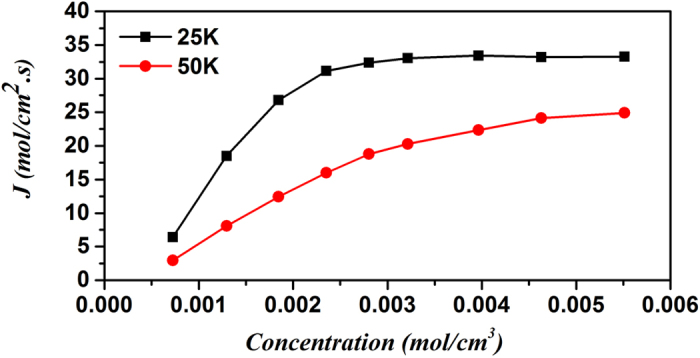
The average concentration dependence of the unit diffusion flux of helium. The angle of asymmetric channel is 38.9°, which refers to the corresponding carbon nanotubes in Fig. 2.

Apart from factors of angle, temperature and atomic attraction, the aperture of the asymmetric channel can also influence the molecular pump effect. According to Fig. 4, in the asymmetric channel with an angle of 38.9°, the larger the aperture, the less the pump effect is, and the accompanying peak gradually goes down with its position moving to lower temperature. When the aperture increases to 1.14 *nm*, the peak vanishes. According to Fig. 6, the pumping efficiency increases with a decreasing speed with the increase of the average density of atoms and converges to a constant eventually. The convergent process may vary under different temperatures. In a word, the pumping efficiency has a close relationship with the temperature, angle, atomic attraction, aperture and average density of atoms in system of extremely small size.

## Extended Model

**Figure 7:**
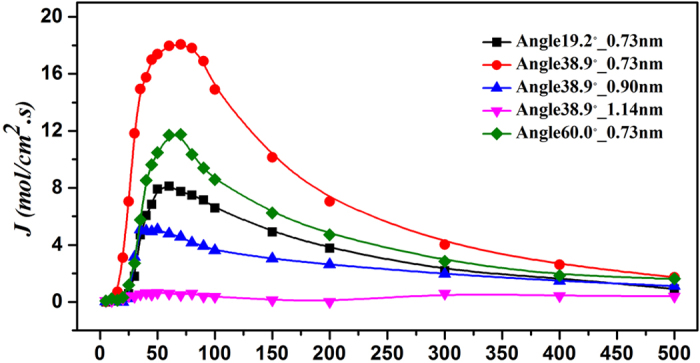
Temperature dependence of the unit diffusion flux of Ne in different asymmetric nanotubes.

According to the previous study, in the asymmetric nanotube with small size, helium shows the directional transport phenomenon. One may wonder if this could happen on the other types of rare gases, i.e. if this phenomenon is universal among rare gases. To address this question, we perform an analogous simulation on neon atom, where the concentration of both the left and right atom reservoirs is 0.0015 *mol/cm*^3^ and some specific results are depicted in Fig. 7. Aside from that all the temperatures (60 K) corresponding to their peaks are higher than that (30 K) of helium, the peak of the pump effect in neon moves to the lower temperature as the apertures increases, especially in the channel with angle of 38.9°. This result validates that the directional transport movement in tiny asymmetric channel for rare gas might be a universal phenomenon which is closely connected with factors such as temperature, aperture, angle, attraction and concentration.

## Efficiency of the Molecular Pump

**Figure 8:**
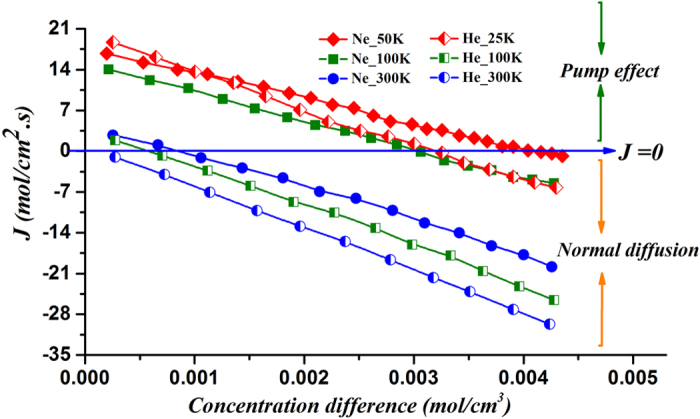
The right side concentration difference dependence of the unit diffusion flux with left side concentration fixed; normal diffusion and pumping effect mean the particle flux is along or against with the concentration gradient, respectively.

After proving the validity of the phenomenon, we are going to measure about the pumping efficiency. Here the transport channel is the asymmetric carbon nanotube with an angle of 38.9°, and the concentration of theleft atom reservoiris 0.0015 *mol/cm*^3^, while the concentration of theright atom reservoir can be adjusted from 0.0015 to 0.0060 *mol/cm*^3^. It can be observed in Fig. 8 that the asymmetricchannel can pump helium against a concentration gradient of 0.003 *mol/cm*^3^ at 25 K. Similar results for neon are also included. It can be deducted that under proper conditions both He and Ne can generate power strong enough to offset the concentration gradient, however, the concentration gradient that the channel can overcome decreaseswith the temperature.

**Figure 9:**
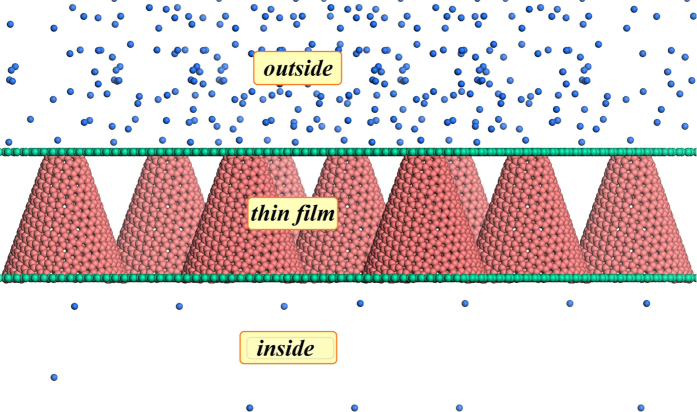
Asymmetric nano thin film for pumping vacuum.

We have to mention that the asymmetric channel is not only for pumping helium and neon, others fluid systems such as O_2_, CO_2_, CH_4_ and air can also be pumped through this structure. The only difference might be the aperture of nanochannel. In order to approach to the application, we assume a nanoscale thin film as shown in Fig. 9, which is composed of many asymmetric channels as displayed in Fig. 1. If we use this structure as the wall of a container, the gases inside the container will flow outside. According to the concentration gradient it can overcome, we can approximately calculate the pressure difference between inside and outside from the state equation of ideal gas, PV = nRT, where P is the pressure, V is the volume of the container, n is the mole density, R is the gas constant and T is the temperature. The maximum pressure difference that can be pumped against reaches to 3.19 atm for He and 92.3 atm for Ne, respectively. According to this result, pumping vacuum will be an easy work just like capillarity.

Here we have to mention that the autonomous pumping depends strongly on the size of asymmetric carbon nanotubes. In another word, it only works wellfor nanostructures, which can generate a susceptible force field. As shown in Figs 4 and 7, when the size of the aperture increases, the pumping effect will decrease and disappear in the end.

## Conclusions

In extremely small size systems, one can use the asymmetric carbon nanotubes to realize the molecular pump, which is explained that in the area of positive force more atoms resistthe thermal disturbance. In that procedure, one has to take into account several factors related with pump effect such as the angle of the carbon nanocone, the atomic attraction from the wall of the channel, the apertures of both entrances of the channel, the temperature of simulation, the average concentration of atoms, the type of rare gases and so on. Most interestingly, we display a greenpath to pumping vacuum without consume any energy. Our results also provide an optional way for studying the molecular transport in bionics and organism.

## Additional Information

**How to cite this article**: Xu, Z.-c. *et al.* Autonomous pump against concentration gradient. *Sci. Rep.* **6**, 23414; doi: 10.1038/srep23414 (2016).
